# Penile skin flap: a versatile substitute for anterior urethral stricture

**DOI:** 10.1590/S1677-5538.IBJU.2018.0652

**Published:** 2019-01-29

**Authors:** Wissem Hmida, Mouna Ben Othmen, Amidou Bako, Mehdi Jaidane, Faouzi Mosbah

**Affiliations:** 1 Department of Urology Sahloul Hospital Sousse Sousse Tunísia Department of Urology, Sahloul Hospital Sousse, Sousse, Tunísia

**Keywords:** Penis, Urethral Stricture, Bulbourethral Glands

## Abstract

**Purpose:**

Penile skin flap uretroplasty is a useful technique for a long urethral stricture due to the ample length and surgical handling characteristics. We investigated the surgical technique and initial results of uretroplasty for anterior urethral strictures using a dorsal penile skin flap.

**Patients and methods:**

From January 2003 to January 2018, a total of 77 patients underwent substitution urethroplasty using dorsal penile skin flap for bulbar urethral strictures in our institution. All patients were assessed preoperatively, and followed postoperatively by physical examination, urinalysis, retrograde and voiding urethrography, uroflowmetry and post-void residual urine measurement. Success was defined as no requirement of additional urethral instrumentation.

**Results:**

The mean age was 45 years (10-87). The mean stricture length was 5cm (3-10cm). The mean flap length was 6cm. Urinary fistula was the most common postoperative complication. The mean follow-up was 60 months (6-120). The overall success rate was 88%. Recurrent strictures were found in 4 patients (5%) at 1 year. At 3 year follow-up, 5 (7%) more patients had recurrences. All recurrences were managed by internal urethrotomy.

**Conclusions:**

Substitution urethroplasty using penile skin flap appear to be a safe and efficient technique for the treatment of a long and complex anterior urethral stricture. It provides encouraging cosmetic and functional results.

## INTRODUCTION

Urethral stricture disease is a heterogeneous condition that often requires a wide array of surgical techniques for a successful repair. Treatment options include dilation, urethrotomy and reconstructive surgical techniques (1).

As clearly evident in the literature, isolated and short bulbourethral strictures inferior to 1.5-2cm are treated by excision and anastomotic repair. On the other hand, complex anterior urethral reconstruction relies on a tissue transfer technique in the form of either a free graft and/or pedicled flap (2). The challenge lies in choosing the appropriate technique for a particular stricture (3, 4). Both are successful individually (5, 6). Penile skin flap can be used in long anterior urethral strictures. Its adaptability comes from its mobile, well-vascularized pedicle and elastic skin island that can be used from the membranous urethra to the fossa navicularis.

In the current era of buccal mucosa, now considered the donor substitute of choice for augmentation, the success of the penile skin flaps in the management of anterior urethral strictures should be reassessed.

The objective of this retrospective study is to describe surgical technique, indications and initial results with dorsal penile skin flap for anterior urethral strictures.

## PATIENTS AND METHODS

### Patients

After obtaining approval from our ethical committee (n° 212/18), we retrospectively identified a total of 77 patients (from January 2003 to January 2018) who underwent urethroplasty using dorsal penile skin flap for bulbar urethral strictures in our institution. Patients with lichen sclerosus and failed hypospadias repair were excluded.

All patients were evaluated preoperatively by physical examination, urinalysis, uroflowmetry and retrograde and voiding urethrography. In all patients, a dorsal onlay flap urethroplasty was performed using penile skin flap. Success and failure was defined respectively by the absence of or the need for any subsequent urethral procedure (dilation, internal optical urethrotomy or repeat open urethroplasty). Postoperative complications were recorded and classified as early (onset: 30d) or late (onset: > 90d) complications, depending on the date of onset. They were also graded according to the modified Clavien system (7).

All patients were followed at 1 month postoperatively, followed by 3-month intervals for the first year and annually thereafter. Follow-up consisted of physical examination, urinalysis and uroflowmetry. In case of suspicion of recurrence (clinically or on uroflowmetry: Qmax < 15mL/s), a retrograde urethrography or urethroscopy was done. Statistical analysis was conducted using chi-square and Student t tests.

### Surgical technique

The operation was made under spinal anaesthesia. The patient is placed in the lithotomy position. A foley catheter is inserted through the urethra until the stricture. A midline perineal incision is made. The urethra is completely mobilized from the corpora cavernosa. Next, it is rotated 180 degrees, and is incised along its dorsal surface. The stricture is opened along its whole length.

**Figure 1A:**
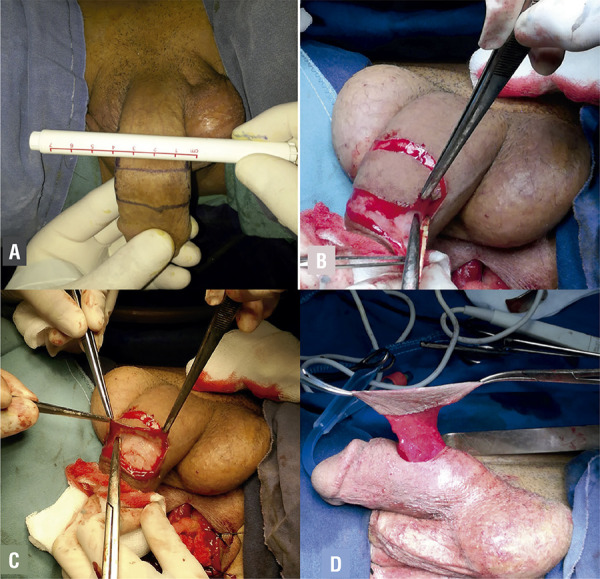
The flap width is carefully measured using a surgical skin marker; Figure 1B - The flap is raised; Figure 1C - The flap is dissected to an extent; Figure 1D - Final aspect of the flap.

For all patients we use dorsal penile skin for urethral reconstruction. The flap width is carefully measured using a surgical skin marker (Figure-1A). In order to avoid redundancy and succulations, the flap width should not exceed 20mm. The flap length is measured using the current formula: L = US (urethral stricture) + (US*0.2) (8). Then, the flap is raised (Figure-1B). The pedicle is mobilized proximally to an extent that allows ventral transposition of the flap without tension (Figure-1C). The dorsal penile neurovascular complex and tunica albuginea are exposed and preserved immediately beneath the plane of dissection. The superficial lamina of Buck’s fascia is elevated with the pedicle flap, thereby supplying its foundation (Figure-1D).

**Figure 2A:**
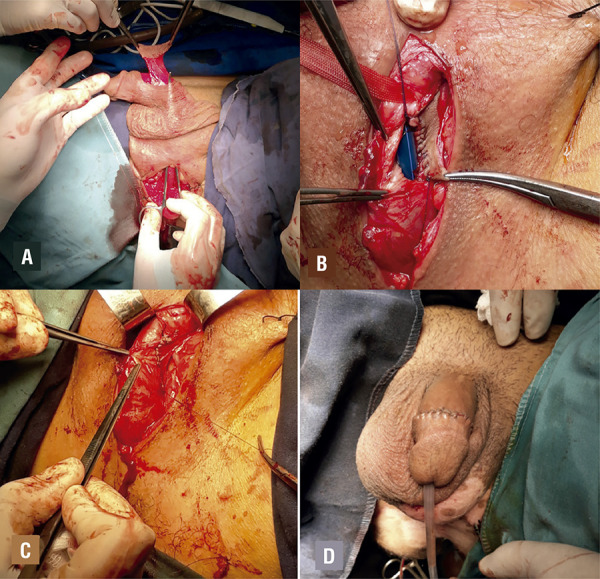
The flap is passed through a scrotal tunnel to the bulb without torsion and without placing excessive tension on the pedicle; Figure 2B - The flap is sutured to the adjoining edges of urethra; Figure 2C - Final aspect of urethra, Figure 2D - Final aspect of the penis.

The flap is passed through a scrotal tunnel to the bulb without torsion and without placing excessive tension on the pedicle (Figure-2A). It is then brought on to the exposed dorsally opened portion of the urethra and sutured to adjoining edges of the urethra using continuous fine sutures over a 16-F Foley catheter (Figures 2B-D). Foley catheter and suprapubic catheter, if placed preoperatively, are left indwelling for 14 weeks. A suction drain is placed on the contra lateral side of the pedicle and removed on postoperative day 2. A retrograde and voiding urethrography is performed at the time of catheter removal in all patients. Any extravasation is managed by extending the period of catheterization.

## RESULTS

The mean age was 45 years (10-87). The etiology of stricture was infectious in 40 (52%), traumatic in 28 (36%) and iatrogenic in 9 (12%). Only 8 (11%) are primary, 69 patients (89%) had a history of urethral intervention in the form of dilatations, internal urethrotomy (IU) or urethroplasty (Table-1). The stricture was located at the bulbar urethra in 70 cases (90%), perineal urethra in 5 cases (7%) and at the penile urethra in 2 cases (3%). The mean stricture length was 5cm (3-10cm). The mean flap length was 6cm (4-10cm). Urinary fistula was the most common postoperative complication. Postoperative complications are listed in Table-2.

**Table 1:** Preoperative parameters.

Parameters	Value
Age (y), mean	45 (10- 87)
Procedures performed previously, n (%)	69 (89%)
Internal urethrotomy	50 (65%)
Urethroplasty	15 (19%)
Urethral calibration	4 (5 %)
**Etiology, n (%)**	
Infectious	40 (52%)
Traumatic	28 (36%)
Iatrogenic	9 (12%)
**Stricture location, n (%)**	
Bulbar	70 (90%)
Perineal	5 (7%)
Penile	2 (3%)
Stricture (cm), mean	5 (3-10)

**Table 2:** Operative and follow-up data.

Parameters		Value
Flap length (cm), mean		6 (4-10)
**Early Complications, n (%)**	Clavien Grade	9 (12%)
Urethrocutaneous fistula	I	4 (5%)
Orchiepididymitis	IIS	2 (3%)
UTI	II	2 (3%)
Hematoma	I	1 (1%)
Recurrence at 1 year, n (%)		4 (5%)
Recurrence at 3 year, n (%)		5 (7%)
The overall success rate, n (%)		68 (88%)

**UTI** = Urinary tract infection

The mean follow-up was 60 months (6-120). Recurrent strictures were found in 4 patients (5%) at 1 year. At 3 year follow-up, 5 (7%) more patients had recurrences. All recurrences were managed by internal urethrotomy. The overall success rate was 88%.

## DISCUSSION

No single approach is appropriate for all urethral strictures. Many different reconstructive techniques have been described and are chosen based on the length, location and extent of spongiofibrous tissue contributing to the stricture. While, end to end uretroplasty is appropriate for short stricture with a high success rate, the use of flaps or grafts is mandatory in patients with longer and complex strictures (9, 10).

The controversy over the best means of reconstructing the urethra, using ﬂap or graft, is still under debate. The current literature, however, does not clearly support the use of one technique over the other (11). In the late of 1990s, buccal mucosa became a standard for reconstructing urethral strictures due to its advantageous histological properties and high success rate (12). However, several disadvantages should be considered. It requires the need of an additional operation field and additional specialised nursing and/or surgical personnel for oral graft harvest (13). Moreover, various donor sites related complications are reported including, oral pain, oral tightness and alterations in saliva production (14, 15).

We observed that many experienced urologists are unfamiliar with the use of penile skin of urethral reconstruction. Although, penile skin flap is our preferred reconstructive technique, because of its excellent cosmetic and functional outcomes.

Penile skin has become a good urethral substitute because of ease of harvest, surgical handling characteristics, hairlessness, and compatibility in a wet environment. On the other hand, it has a flexible tissue with a rich vascular supply allowing to reconstruct long and complex urethral strictures as in our patients.

In our institution, we have adopted this technique for 14 years and we consider that harvesting of penile skin is safe and technically simple for all urologists, as it requires no special experience with oral surgery or knowledge of oral anatomy (13).

Over viewing the published reports, there is strong evidence that substitution urethroplasty using penile skin flaps has acceptable results.

Most authors have reported identical success rates for both buccal mucosa graft and penile skin flaps (16, 17). In a retrospective analysis of 299 patients, Fu Q et al. (18) reported a similar success rate with buccal mucosa (85%) as compared with penile skin flap (83%). Similarly, in a comparative study including 69 patients, the success rate was equal for both buccal mucosa graft (90%) and penile skin flap (84%) (19).

Alsikafi et al. compared the outcome of 95 buccal mucosa urethroplasty and 24 penile skin flap urethroplasty. The overall success rate of penile skin urethroplasty was 84% in a mean follow-up of 201 months (20). Similarly, Dubey et al., reported on 28 patients who underwent longitudinal penile skin flap a success rate of 85%. In this study, the stricture recurrence was described in 4 patients (17). Quartey et al., reported with transverse preputial or penile flap a success rate of 99% (21).

In our study, the overall success rate was 88%. Surgical failure was reported in 12% of our patients, wherein focal recurrence occurred mainly at the anastomotic margin showing results similar to those of previous studies (22, 23).

The best location for placing grafts (ventral or dorsal) remains controversial. Some authors, have suggested that ventral placement of the flap/graft can lead to complications like urethral diverticulum formation and succulations with postvoid dribbling and ejaculatory failure (24, 25). However, some others have reported good long-term stricture-free outcomes equal to dorsal onlay using this technique (6, 26). In our series, the transverse penile skin flaps were fashioned and rotated to be dorsally quilted into the dorsally opened strictured part of the urethra without vascular compromise. Based in our experience, it seems that the outcomes of this technique are encouraging.

A review of literature showed a variability of rate and type of complications reported. The rate of occurrence of urethrocutaneous fistula ranges from 0 to 13% (3, 17, 23). In the present study, only 4 patients (5%) developed urethrocutaneous fistula.

The incidence of pseudodiverticulum formation in flap repair ranges from 0 to 5% in various series (17, 27, 28). In our series, the incidence of pseudodiverticulum formation was 0%. In this respect, dorsal onlay flaps are more advantageous than ventral placement. Necrosis of the penile skin was less reported. It results when the vascular supply of the sub-dermal plexus was compromised. This is an inherent disadvantage of any pedicle penile skin flap, although in experienced hands its incidence is lower (24). In the present study, no case of penile skin necrosis has been reported.

Any kind of substitution urethroplasty deteriorates over time. Long-term results with skin flap urethroplasty show a decreasing success rate with time. Peterson et al. demonstrated in a multicenter study a higher failure rate when longer follow-up was considered (18.4% at 58.8 months). The high rate of recurrence can be attributed to a poor urethral quality. In our series, urethral stricture was reported in 9 patients (12%).

## CONCLUSIONS

Substitution urethroplasty using penile skin flap appears to be a safe and efficient technique for the treatment of a long and complex anterior urethral stricture. It provides encouraging cosmetic and functional results.
